# Mechanically reinforced core-shell scaffold with integrated structure and function for accelerated tendon repair

**DOI:** 10.1093/rb/rbaf088

**Published:** 2025-08-18

**Authors:** Xiaoxi Long, Yanzhao Dong, Ting Guo, Yiting Zhang, Peng Liu, Yongpeng Wu, Hui Lu, Xianwei Wang, Hemin Nie, Swee Hin Teoh, Feng Wen, Zuyong Wang

**Affiliations:** College of Materials Science and Engineering, College of Biology, Hunan University, Changsha 410082, China; Department of Orthopedics, The First Affiliated Hospital of Zhejiang University, Hangzhou 310003, China; College of Materials Science and Engineering, College of Biology, Hunan University, Changsha 410082, China; AECC Guizhou Liyang Aviation Power Co., Ltd, Guiyang 550014, China; College of Materials Science and Engineering, College of Biology, Hunan University, Changsha 410082, China; College of Materials Science and Engineering, College of Biology, Hunan University, Changsha 410082, China; College of Materials Science and Engineering, College of Biology, Hunan University, Changsha 410082, China; Department of Orthopedics, The First Affiliated Hospital of Zhejiang University, Hangzhou 310003, China; Department of Vascular Surgery, Xiangya Hospital, Central South University, Changsha 410008, China; College of Materials Science and Engineering, College of Biology, Hunan University, Changsha 410082, China; College of Materials Science and Engineering, College of Biology, Hunan University, Changsha 410082, China; Zhejiang Engineering Research Centre for Tissue Repair Materials, Wenzhou Institute, University of Chinese Academy of Sciences, Wenzhou 325001, China; College of Materials Science and Engineering, College of Biology, Hunan University, Changsha 410082, China

**Keywords:** tissue engineering, tendon, scaffold, core-shell structure

## Abstract

Core-shell scaffold designs that mimic the biophysical structure of tendon extracellular matrix offer unique advantages for tendon repair. However, balancing the structural integrity of the scaffold with the desired material and biological properties remains challenging, limiting the effectiveness of the scaffold. Here, we present a new method for fabricating a core-shell scaffold with tailored properties for tendon tissue engineering. The scaffold core, designed for cell guidance, was created using direct ink writing, resulting in a helically interconnected fibre structure with controllable anisotropy and pore sizes. The mechanically reinforced shell, produced through uniaxial cold stretching of a laser-drilled sheet, featured microsurface ridges and through-hole arrays. The core-shell integration enabled sequential degradation and mechanical properties aligned with tendon tissue requirements, providing extended structural support and improved space for neotissue ingrowth. *In vitro* and *in vivo* studies confirmed the scaffold’s non-cytotoxicity and superior tendon matrix regeneration, with increased collagen deposition and structural alignment compared to controls. These findings highlight the potential of the developed scaffold for advancing tendon repair applications.

## Graphical Abstract

**Figure:**
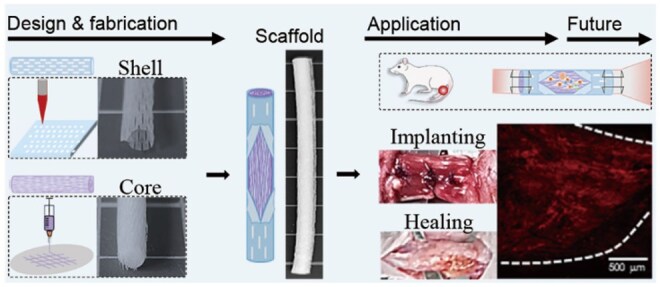

## Introduction

Tendons are essential components of the musculoskeletal system, connecting muscles to bones to transmit forces generated by muscle contraction and buffer stress to prevent concentration. This makes tendons susceptible to damage under substantial tensile loading. Globally, tendon ruptures affect 80–90 individuals per 100 000 annually, impacting 6–7 million people [1]. In the USA, flexor tendon repair costs approximately $13 725 per patient, amounting to $409.1 million annually [2]. Unlike the bones and muscles, tendons have limited regenerative capacity due to poor cellularity and vascularity. In addition, the human Achilles tendon, e.g. experiences a large variation in the local strains and exhibits a range of high Young’s modulus from 217 to 1200 MPa [3]. Consequently, tendon injuries can lead to prolonged disability and weaker, damage-prone tissue. Mild injuries are typically managed with conservative treatments such as R.I.C.E. (Rest, Ice, Compression, Elevation), brace support, and/or anti-inflammatory injections [4]. If untreated, severe injuries, including partial or complete lacerations, can result in gapping, compromising tendon structure and function [5]. To address the gap, the concept of tissue-engineered scaffolds was proposed, which includes synthetic and natural scaffolds such as the decellularized tissues. GraftJacket^®^ as one of the commercialized decellularized tissues, has been developed for Achilles tendon repair, demonstrating the potential of tissue-engineered scaffold in advancing tendon treatment [6]. However, decellularized tissue may pose risks of disease transmission and immune rejection. Synthetic scaffolds, by contrast, offer well-defined properties, including controlled molecular weight, degradation rates and mechanical characteristics, without genetic or antigenic residues.

The morphology of tissue-engineered scaffolds for tendon defect includes 2D and 3D fabricated from hydrogel [7], braided [8], knitted [9], multi-layered [10], multi-compartmental [11] and core-shell [12] designs. Besides the morphology of the scaffold, mechanical characteristics and biological performance are equally important. Tendons transmit the strength of muscles to bones, thus enabling movement. Therefore, load-bearing scaffolds provide the necessary mechanical support for tendon regeneration, while the delivery of bioactive factors promotes the biological processes of cell growth and tissue formation. Successful tendon scaffolds typically combine these two aspects, mimicking the mechanical properties of natural tendons and delivering growth factors to facilitate tendon defect healing. Aligned microtopography has been shown to modify patches to mimic the anisotropic structure of tendon extracellular matrix (ECM) [13]. Hydrogels are generally considered to possess properties similar to native ECM, making them effective for drug delivery. However, their mechanical properties are inferior to those of ligaments and tendons [14]. Braided [15], knitted [16] and core-shell [17] scaffolds offer exceptional mechanical strength. In addition to the aforementioned methods, some new approaches for the study of tendon defects have also been developed, such as 3D hexagonal weaving technology [18], hydrogel-nanofibre composites [19], smart stimulus-responsive systems [20], and 3D bioprinted tendon chip models [21]. Among the advances, the core-shell scaffold design could achieve up to 94% porosity and meet the mechanical strength requirements (e.g. a Young’s modulus of 375 MPa) of tendons [17]. By optimizing material composition and structural design, core-shell scaffolds can effectively mimic the architecture of tendon ECM, balancing bioactivity with tissue regeneration [22]. Additionally, core-shell scaffolds can integrate compartmental designs to further replicate the hierarchical structure and compartmentalization of tendons [23].

Historically, core-shell structures have been used in tissue engineering, particularly for fibres and scaffolds. For example, core-shell fibres at the nanoscale were fabricated into films with microRNA encapsulated in the core and polylactide-polyethylene glycol copolymers serving as anti-adhesive shells [24]. Similarly, core-shell fibres have been fabricated using coaxial electrohydrodynamic (EHD) printing to create a controllable architecture [25]. However, in tendon tissue engineering, the fibres often exhibit inadequate mechanical properties. To address this, 3D scaffolds with core-shell structures have been developed to enhance mechanical strength for tendon applications [26]. These scaffolds incorporate an anisotropic collagen-glycosaminoglycan (CG) porous core and a dense, isotropic CG membrane shell to facilitate osmotic transport while maintaining adequate stiffness (24 MPa). Further research has modified the CG membrane shell to include well-ordered arrays of microscale perforations, improving nutrient transport and promoting cell migration [27].

On the other hand, for promoting beneficial biological responses, decellularized amniotic matrix has been reported to encapsulate the traditional collagen-chondroitin sulphate structure, forming an immunomodulatory core-shell scaffold [28]. By microinjecting human mesenchymal stem cells (MSCs) into printed poly(ε-caprolactone) (PCL) tubes, a composite tendon construct was formed featuring a core-shell structure design [12]. This strategy overcame the limitation of low cell density in traditional cell-seeding scaffolds, achieving anatomically relevant cell density through the use of aliphatic polyesters or bioprinted cell-laden hydrogel constructs. Our method provides an alternative to enhance the scaffold’s mechanical properties, cell migration and ingrowth, using a core portion surrounded by a dense membrane and integrated fibrous shell created via direct EHD jet printing. To replicate the native tendon, the tendon graft needs to meet three critical mechanical criteria, including the low creep (e.g. <5% strain under prolonged load) to prevent gradual elongation and loss of function, controlled failure strain (<15%) to avoid dangerous overstretching before rupture, and nonlinear elasticity matching mimicking the toe region (low-strain flexibility) and linear region (high-strain stiffness) of natural tendon stress–strain curves. Aligned electrospun fibres can mimic tendon’s crimp structure, and core-shell structures facilitate both nonlinear elasticity matching and control of failure strain. We integrated single-axial stretching with electrospinning and laser perforation to produce a perforated tubular shell surrounding the scaffold core, with longitudinally aligned fibres [17]. In a minipig model, the scaffold demonstrated high tensile strength while retaining tendon-like properties, effectively promoting cell alignment and tendon tissue regeneration.

While tendon scaffolds with core-shell structure provide unique mechanical benefits and modular biofunctional design, the fabrication involves complex manufacturing parameters, and it is difficult to achieve precise control of the structure (e.g. single fibres) at the cell-relevant microscales. This, for example, can lead to the structural collapse of the fibrous core after implantation, resulting in reduced cell infiltration and inhomogeneous tendon regeneration. Current solutions have not fully addressed challenges associated with the core-shell tendon scaffolds. Here, we present a novel approach for fabricating core-shell scaffolds that enables precise structural control and optimization of key properties for tendon tissue engineering. The scaffold possesses a unique 3D architecture that shows sequential core-to-shell degradation, improved surface wettability and mechanical properties approaching the human Achilles tendon. *In vitro* and *in vivo* studies have confirmed that the scaffold is non-cytotoxic and has the ability to promote tendon regeneration, facilitating aligned matrix fibre formation and structural organization. This work shows promise in overcoming the long-standing challenges in the structure-property relationship of tendon scaffolds, with the potential to advance tendon repair strategies.

## Materials and methods

### Materials

PCL (Mn = 80 000), sodium dodecyl sulphate and fluorescein diacetate (FDA) were purchased from Sigma-Aldrich Co., Ltd (USA). Triton X-100, Hematoxylin-Eosin Staining Kit (G1120), Sirius Red Staining Kit (G1472) and Masson’s Trichrome Staining Kit (G1340) were purchased from Beijing Solebo Technology Co., Ltd (China). Ethanol (EtOH), sodium hydroxide (NaOH) and dichloromethane (DCM) were purchased from Sinopharm Chemical Reagent Co., Ltd (China). Foetal bovine serum (FBS), trypsin, and Dulbecco’s Modified Eagle Medium (DMEM) were purchased from Gibco Co., Ltd (USA). Propidium iodide (PI), phosphate buffer saline (PBS) and bovine serum albumin (BSA) were purchased from Dalian Meilun Biotechnology Co., Ltd (China). The CCK-8 kit (CCK-8) was purchased from Shanghai Tao Zhi Biotechnology Co., Ltd (China). TRIzol^TM^ solution, Alexa Fluor^TM^ 594 Goat Anti-Mouse IgG (H + L) and cell culture flasks were purchased from Thermo Fisher Scientific (USA). HiScript^®^ III RT SuperMix for qPCR (+gDNA wiper) and ChamQ Universal SYBR qPCR Master Mix were purchased from Vazyme Biotech Co., Ltd (China). Collagen type I primary antibody (COL-I, SC-59772) was purchased from Santa Cruz Biotechnology, Inc. (USA). Paraformaldehyde solution (PFA) was purchased from Biosharp (China). Antifade mounting medium with 4,6-diamidino-2-phenylindole (DAPI) was purchased from Solarbio Science & Technology Co., Ltd (China). Cell culture plates (24-well) were purchased from SuRui (China). Centrifuge tubes (5 mL, 10 mL) and pipettes (75 mL) were purchased from Sorfa Life Science Research Co., Ltd (China). Surgipro sutures of size 5-0 were obtained from COVIDIEN Co., Ltd (USA).

### Fabrication of core-shell scaffold

PCL solution (30 wt.%) was prepared as the ink using a mixed DCM/EtOH (7:3, v/v) solvent. The solution was fabricated into fibrous sheets at a pre-designed geometry using consistent parameters of needle size (21 G), applied voltage (3 kV), feed rate (20 µL min^−1^), tip-to-collector distance (2.5 mm) and stage speed (80 mm s^−1^). The as-fabricated sheet is denoted as PCL_Fb_ at different fibre angles of 15° and 90°.

To fabricate the scaffold shell, PCL was first prepared as a film by two-roll milling followed by heat pressing. The obtained film is denoted as PCL_Fm_. This film was drilled using a femtosecond laser (wavelength of 800 nm and frequency of 1000 Hz) to create the penetrated pores. The porous film was named P-PCL_Fm_ and was further rolled and heat-sealed into tubes with a diameter of ∼3 mm. By subjecting it to uniaxial stretching (HYC-2011, Hongjin, China) with a draw ratio of 5 and a stretching speed of 10 mm min^−1^ at room temperature, the PCL tube was fabricated into the shell and named UXP-PCL_Fm_.

The PCL_Fb_ sheet was then rolled into a rod-like core. The core-shell tendon scaffold was obtained by inserting the core into the shell. The weight of the scaffold was labelled as W_s_, and the weight calculated based on the volume of the scaffold and a density of PCL (1.145 g mL^−1^ at 25°C) was labelled W_c_. A porosity of the scaffold was calculated as follows:


(1) $$\mathrm{Porosity}\;\left(\%\right)=100\%-\left(W_{c}-W_{s}\right)/W_{c}\times 100\%$$

### Material characterizations

Scanning electron microscopy (SEM): Samples were gold-sputtered at 6 mA for 90 s before imaging using an SEM (MIRA3 LMN, TESCAN, Czech Republic). Images were acquired at 5 kV at random locations on the samples. To observe the cross section of the scaffold, samples were prepared with brittle fracture in liquid nitrogen.

Fourier transform infrared spectroscopy (FTIR): Samples were fixed on a flat diamond testing bench. Analysis was conducted utilizing an FTIR spectrometer (Nicolet iS50, Thermo Scientific, USA). The wavenumbers examined ranged from 400 to 4000 cm^−1^. Functional groups were identified by comparing the obtained spectra with the previously reported [29].

X-ray diffraction (XRD): Samples were cut into 1 × 1 cm^2^ pieces and examined by XRD (MINIFLEX600, Rigaku, Japan) using Cu-Kα radiation under atmospheric conditions. Data were collected at a step width of 0.2°, duration of 1.2 s, wavelength of 1.5418 Å, and 2θ values of 5–90°. The phase structure was identified by comparing the diffraction patterns with the ICDD standards (JCPDS) [30]. The average grain size of PCL was estimated by the Scherrer Method [31]:


(2) Grain size (nm)=κ×λ/β×cos θ

where κ, λ, β and θ represent the constant shape factor (0.9), X-ray wavelength, the width at half maximum and the diffraction angle, respectively.

Thermogravimetric analysis (TGA): Thermal stability (10 mg per sample) was performed using a thermogravimetric analyser (STA 2500, NETZSCH, Germany). Testing was performed under argon gas flow (100 mL min^−1^), at a heating speed of 10°C min^−1^ from room temperature to 600°C. A derivative thermogravimetric curve was obtained by first-order derivative processing of the TGA curve.

Differential scanning calorimetry (DSC): Material melting and crystallization were determined using DSC (DSC 300, NETZSCH, Germany). Testing was performed in an air environment with a temperature range of 20–90°C and heating rate of 10°C min^−1^. The crystallinity of the scaffold was determined by comparison with the melting enthalpy (139.5 J g^−1^) of 100% crystalline PCL [32].

Water contact angle (WCA): Surface wettability was examined using the built-in function of a contact angle analyser (SDC-200S, ShengDing, China). A 2-µL water droplet was used. Images were taken at 0, 2, 4, 6, 8 and 10 min after contact with the sample surfaces. For anisotropic samples, testing was performed along and across the surface structures. For each group, six samples were used, and at least three random locations of each sample were measured.

Mechanical testing: The rod-like testing samples were prepared at a length of 1 cm and a diameter of 3 mm. By pressuring the sample into a rectangular shape, the thickness was measured using a digital micrometre (0–25 mm, Meinaite, Germany). Testing was performed at a loading speed of 10 mm min^−1^ and a force of 100 N using a tensile testing machine (HYC-2011, Hongjin, China). Data were analysed using the Origin software (Origin9.1, OriginLab, USA). For the sample exhibiting a yield phenomenon, a low yield point was employed to ascertain its yield stress and strain. For the left side, an offset strain of 0.5% was used to determine the yield stress and strain. Five samples from each group were analysed.

Degradation testing—Samples at 1 cm in length and 3 mm in diameter were prepared and immersed in 10 mL of NaOH solution (3 mol L^−1^) at room temperature. The initial weight of the sample was denoted as W_0_. Optical images were captured at each time point before the solution was removed. The samples were washed three times with distilled water and dried for weighing (W_d_). The degree of sample degradation was evaluated based on the mass loss determined as follows:


(3) $$\mathrm{Mass}\;\mathrm{loss}\;\left(\%\right)=\left(W_{0}-W_{d}\right)/W_{0}\times 100\%$$

At each time point, five samples of each group were analysed.

### Cells and culture

Human foetal bone marrow MSCs (HUXMF-01001) were purchased from Cyagen Biotechnology Co., LTD (China). The cells were cultured in DMEM (1% PS + 10% FBS) in flasks (Gibco, USA) in a CO_2_ incubator (BB50, Thermo Fisher Scientific, USA) at 37°C. The culture medium was replaced every three days. Cells were digested using 0.25% trypsin for passaging when the cell density reached 90%. For cell studies, all material samples (1 cm in length and 3 mm in diameter) were disinfected by soaking in a 75% EtOH solution for 12 h. For all studies, a 300-µL cell suspension was used for seeding on each sample (seeding density: 30 k/sample). After 6 h of incubation for the initial adhesion, 1 mL of D10 medium was added to each sample. The cells were used before passage 6.

### *In vitro* cell morphology, cytotoxicity and proliferation

The cellular adhesion and morphology were characterized using SEM. Briefly, the cells were cultured for 1 and 14 days after seeding. The cells were fixed with PFA (4 wt.% in PBS, 15 min), dehydrated by gradient EtOH solutions (30, 50, 70, 90, 95 and 100%, 15 min) and then surface gold coated for SEM characterization.

Cytotoxicity was evaluated using live and dead cell staining. After culturing for pre-determined periods, MSCs were incubated with FDA (4 µg mL^−1^ in PBS) and PI (1 µg mL^−1^ in PBS) solutions for 8 and 5 min, respectively. The cells were then washed thrice with PBS and examined using a confocal laser scanning microscope (CLSM, FV1000, Olympus, Japan). Live and dead cells were identified as FDA-labelled green and PI-labelled red, respectively.

The metabolic activity was tested to evaluate cell proliferation. After culturing for 1, 3, 7, 14 and 21 days, the cell medium was removed, and the cells were incubated with CCK-8 testing medium (1:10 dilution in D10) for 2 h at 37°C and 5% CO_2_. The supernatant was analysed with a microplate reader (2300, EnSpire, Singapore) at a wavelength of 450 nm. Four samples from each group were collected at each time point.

### *In vitro* tendon-associated gene and protein expression

Tendon-associated gene expression was determined by a quantitative reverse transcription polymerase chain reaction (qRT-PCR, Q711-02, Vazyme, China). Briefly, MSCs were cultured on the scaffold for 5 and 10 days. PCL_Fm_ and PCL_Fb_ (90°) were used as the controls of the scaffold’s shell and core, respectively. Extraction of total cell RNA, cDNA synthesis and real-time detection were performed using TRIzol^TM^ Solution, HiScript^®^ III RT SuperMix and ChamQ Universal SYBR qPCR Master Mix, respectively. The primers specific to interested genes including scleraxis (Scx), decorin (DCN), tenascin-C (TNC) and COL-I were summarized in Supplementary Table S1 and provided by Sangon Biotech (China) [33]. Relative gene expression level was determined by comparing the quantified cDNA transcript level to that of GAPDH. The expression levels of all groups were then normalized to the group of cells before seeding on materials, which was set as 1. Triplicate detections were performed for each sample, and three samples were used for each group.

The expression of major tendon matrix protein (COL-I) was examined by immunofluorescence staining. Briefly, MSCs were cultured on the scaffold for 5 and 10 days. PCL_Fm_ and PCL_Fb_ (90°) were used as the controls of the scaffold’s shell and core, respectively. After fixation, permeabilization and blocking, the cells were incubated in sequence with mouse-derived anti-human primary monoclonal antibody (1:200 dilution of anti-COL-I in 1 wt% BSA solution) and 594 Goat Anti-Mouse IgG (H + L) antibody (1:200 dilution in 1 wt% BSA solution). Cells were finally incubated with DAPI for nucleus visualization. Images were taken using CLSM with identical parameters for all groups.

### *In vivo* animal studies

Tendon defect model: Animal procedures were approved by the Committee of Animal Experimental Ethical Inspection, Zhejiang University School of Medicine (Ethics Number: 2022-676). Briefly, 8-week-old male rats (320 ± 20 g) were randomly divided into three groups: the tendon defect group (TDG, six rats), the material treatment group (MTG, six rats) and the Sham group (Sham, two rats). Before surgery, all animals were fasted for 12 h, followed by an intraperitoneal injection of sodium pentobarbital (50 mg mL^−1^, 30 mg kg^−1^). To facilitate the operation, the rats were placed in a supine position. A 3-cm mid-lateral incision was made to expose the Achilles tendon. Using sharp scissors, a half-thickness transection of the Achilles tendon was performed, creating a defect measuring 0.5 cm in length. In the MTG procedure, the defect was semi-sutured using the fabricated tendon scaffold, after which the incision was closed using sutures. In the TDG procedure, the defect was left untreated before incision closure. Sham surgery served as a Sham procedure, with no Achilles tendon injury. The Kessler suture method was employed using absorbable suturing lines. For sample collection in each group, half of the animals were euthanized at 4 weeks, and the remaining animals were euthanized at 8 weeks.

Gross observation: After euthanasia, the rats were placed in a supine orientation. A 5-mm incision was made along the right hind leg to fully expose the Achilles tendon. Gross observations of the animal models were conducted by the same surgeon, documenting any changes (including width, swelling, texture, and adhesion to the surrounding skin) in the tendon.

Histological analysis: The harvested Achilles tendon tissues, along with the materials, were fixed in 4% buffered formalin (pH 7.4) and subsequently embedded in paraffin blocks. Four-micrometre slices were prepared and stained using Hematoxylin-Eosin, Sirius Red and Masson’s Trichrome Staining Kits, following the manufacturer’s instructions. Pathological scoring was performed using a light microscope (BX51, Olympus, Tokyo, Japan) by two pathologists who were blinded to the experimental design. Tissue evaluation was based on the Tang score for tendon adhesion assessment and the Soslowsky, Svessen and Cook (SSC) score for tissue regeneration evaluation (Supplementary Table S2) [34].

Second harmonic generation (SHG) characterization: Pathological specimens from each group were imaged using two-photon microscopy (FVMPE-RS, Olympus, Tokyo, Japan) with an excitation wavelength of 850 nm. Images were captured at a resolution of 1024 × 1024 pixels and a pixel resolution of 4 µm through a 420–460 nm filter. SHG fluorescence of collagen fibres was observed in both injured and uninjured regions. Semi-qualitative analysis was conducted using ImageJ (NIH, MD, USA) to evaluate the density, organization and mean grey values (MGV) of the collagen fibres.

### Statistical analysis

Data are expressed as the mean ± SD unless otherwise specified. Statistical comparisons between two groups were performed using Student’s paired test with a two-tailed distribution in Excel (Microsoft Office 2021, USA). A value of *P* < 0.05 is considered to be significantly different.

## Results and discussion

### Design and fabrication of the tendon scaffold

**Figure 1.**
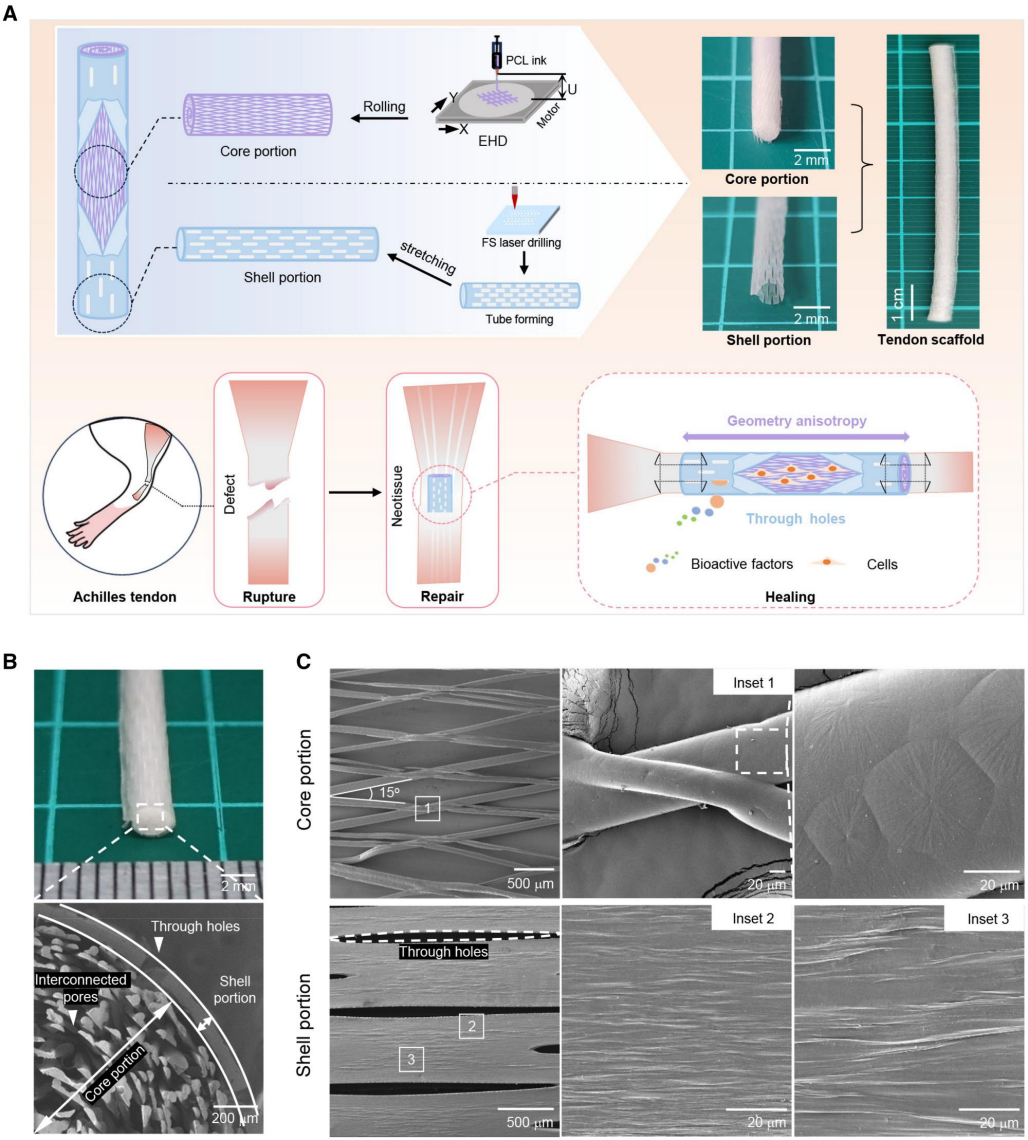
Fabrication of PCL core-shell tendon scaffold. (**A**) Scheme illustrating scaffold design, fabrication and application. Scale bars, 2 mm and 1 cm for optical images. (**B**) Images of the as-fabricated scaffold. Core-shell interface, dashed white line; holes, single-headed white arrow. Scale bars, 2 mm (optical image, top panel) and 200 µm (SEM image, bottom panel). (**C**) SEM images of shell and core surfaces. Inset 1, fibre intersection; inset 2, adjacent surface of the through-hole; and inset 3, surface away from the through-hole. Scale bars, 500 µm (left panel), 20 µm (middle panel) and 20 µm (right panel).

We designed a core-shell scaffold for tendon rupture repair (Figure 1A). The scaffold showed a rod-like core composed of unique fibres for the guidance of cell and matrix alignment. The fibres printed by the EHD technique are tailorable in terms of structural geometry, which allows for controlled porosity and anisotropy. Around the core, a shell structure is designed to provide scaffold mechanical support as well as penetrated channels for nutrition transport and cell and vessel migration from peritendinous tissue. Optical images show a scaffold with an integrated diameter of 3 mm and a length of 70 mm assembled from the core and shell portions.

Figure 1B shows optical and SEM images of the scaffold. It is assembled by inserting the core into the shell and securing it firmly through structural relaxation of the core layers. The space formed by the shell was occupied by the printed fibres, which exhibited numerous interconnected pores that extended into the through holes of the shell. The porosity of this core-shell scaffold was calculated to be approximately 80%. This satisfies the requirement for adequate neotissue ingrowth and may facilitate the transport of essential nutrients and removal of toxic byproducts of cell metabolism.

Additional structural details of the core-shell scaffold are shown in Figure 1C. The fibres of the core are arranged periodically by EHD direct writing, forming rhomboids at intervals of 500 µm and at an angle of 15° as pre-determined. This leads to the well-known geometric anisotropy of scaffolds for contact cell guidance. PCL spheroids were observed on individual fibres, with material consolidation occurring at the interface between the upper and underlying fibres. Concurrently, the upper fibres exhibit morphological adaptability but not complete fusion to the underlying fibres. This permits interconnectivity among the rhomboidal units of the rolled core laminates. In contrast, owing to fibrous segment collapse as observed in existing 3D printed materials, *in situ* continuous layer-by-layer deposition of the upper fibres would segregate the rhomboidal units completely, leading to restricted mass exchange in tissue engineering applications.

SEM images of the shell exhibit multiple surface structures comprising staggered through-holes and non-hole regions with unique micro-ridges/grooves. The direct laser perforation of through-holes resulted in enlarged areas after uniaxial stretching (Supplementary Figure S1). This may have facilitated transverse mass transportation from the shell to the core. Meanwhile, micro-ridges/grooves were observed in the non-hole regions, forming a high geometric anisotropy along the scaffold length (Figure 1C). In our previous studies, it is suggested that mechanical stretching resulted in PCL recrystallization, and the structural deformation caused crystal exposure to the shell surface as ridges and the amorphous regions to depress into grooves [32]. Here, in regions near the through-hole edges, we observed a reduction in the formed inter-ridge-distance, probably because of the experience of a larger Poisson’s contraction [32]. Although the shell exhibits surface micro-ridges/grooves with enlarged through-holes, the nature of the open pores negatively affects the mechanical strength of the scaffold [27]. However, we expect that the strain strengthening induced by uniaxial stretching can compensate for the weakening effect of through-holes.

### Physicochemical characterizations on the tendon scaffold

**Figure 2.**
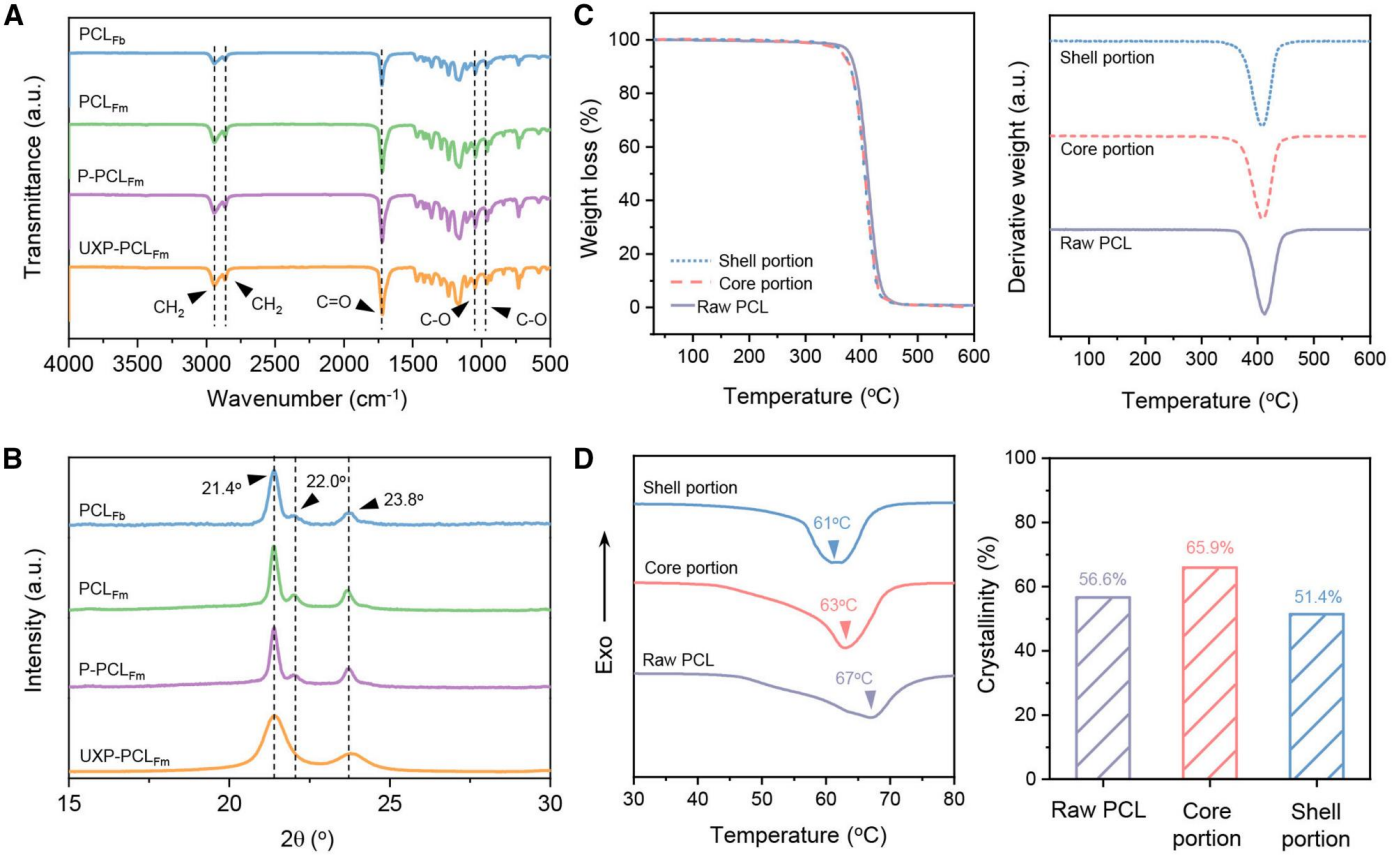
Physicochemical characteristics of the core-shell tendon scaffold. (**A**) FTIR spectra. (**B**) XRD spectra. (**C**) Thermogravimetric (TG) and derivative thermogravimetric (DTG) spectra. (**D**) DSC spectra and calculated crystallinity.

Figure 2A shows the characteristic peaks of C–H, C=O, and C–O stretching vibrations at 2900, 1800 and 1400 cm^−1^, respectively [29]. This indicates that there were no chemical group changes in PCL during the electrohydrodynamic and laser processes. As shown in Figure 2B, the characteristic peaks of the PCL XRD spectrum were observed at 2θ = 21.4°, 22.0° and 23.8°. These peaks correspond to the PCL crystallographic planes of (110), (111) and (200), respectively [30]. Owing to the breakage of the PCL molecular chain by stretching, the characteristic peak at 2θ = 22.0° disappeared in the UX-PCL group, with significantly reduced grain size compared to the other groups (Supplementary Figure S2) [32].

The thermal stability of the scaffold is shown in Figure 2C and Supplementary Figure S2, which show a similar single-step decomposition for all the groups. Compared with raw PCL, the scaffold core and shell exhibited reduced initial decomposition temperatures, suggesting that the fabrication procedures affected the thermal stability of the composite. Consistent with this observation, the scaffold reached maximum decomposition at lower temperatures than raw PCL, further indicating alterations in thermal stability due to the scaffold fabrication processes. This leads to an evident early mass loss in the core-shell scaffold. Further comparisons indicated that the core had better thermal stability than the shell. This may be attributed to uniaxial stretching, which induces molecular chain breakage and thereby lowers the resistance of the material to thermal decomposition.

Figure 2D exhibits the thermo curves of the scaffold core and shell. Compared to raw PCL, the endothermic peak width of PCL became narrow, with reduced melting points observed for the core and shell. Furthermore, the core exhibited a higher degree of PCL crystallinity, indicating enhanced recrystallization of the PCL molecular chains during the EHD writing process of the PCL. This is consistent with the formation of PCL spheroids on the printed fibres (Figure 1C). Conversely, the shell exhibited a decrease in crystallinity (Figure 2D). Our previous study demonstrated that uniaxial stretching of PCL at a temperature close to its melting point, followed by cooling at room temperature, increases PCL crystallinity [32]. In this study, without any thermal treatment, our findings suggest that uniaxial cold stretching may lead to the degradation of PCL molecular chains, resulting in a reduction of the melting point and crystallinity. It is also valuable to note that the melting point of a polymetric material is not solely an intrinsic property, and it is influenced by extrinsic factors such as material structure, environmental conditions, processing history, impurity and experimental setup [35]. This explains why the change in melting point is inconsistent with that in crystallinity, due to the different processing for PCL materials.

### Material properties of the tendon scaffold

**Figure 3.**
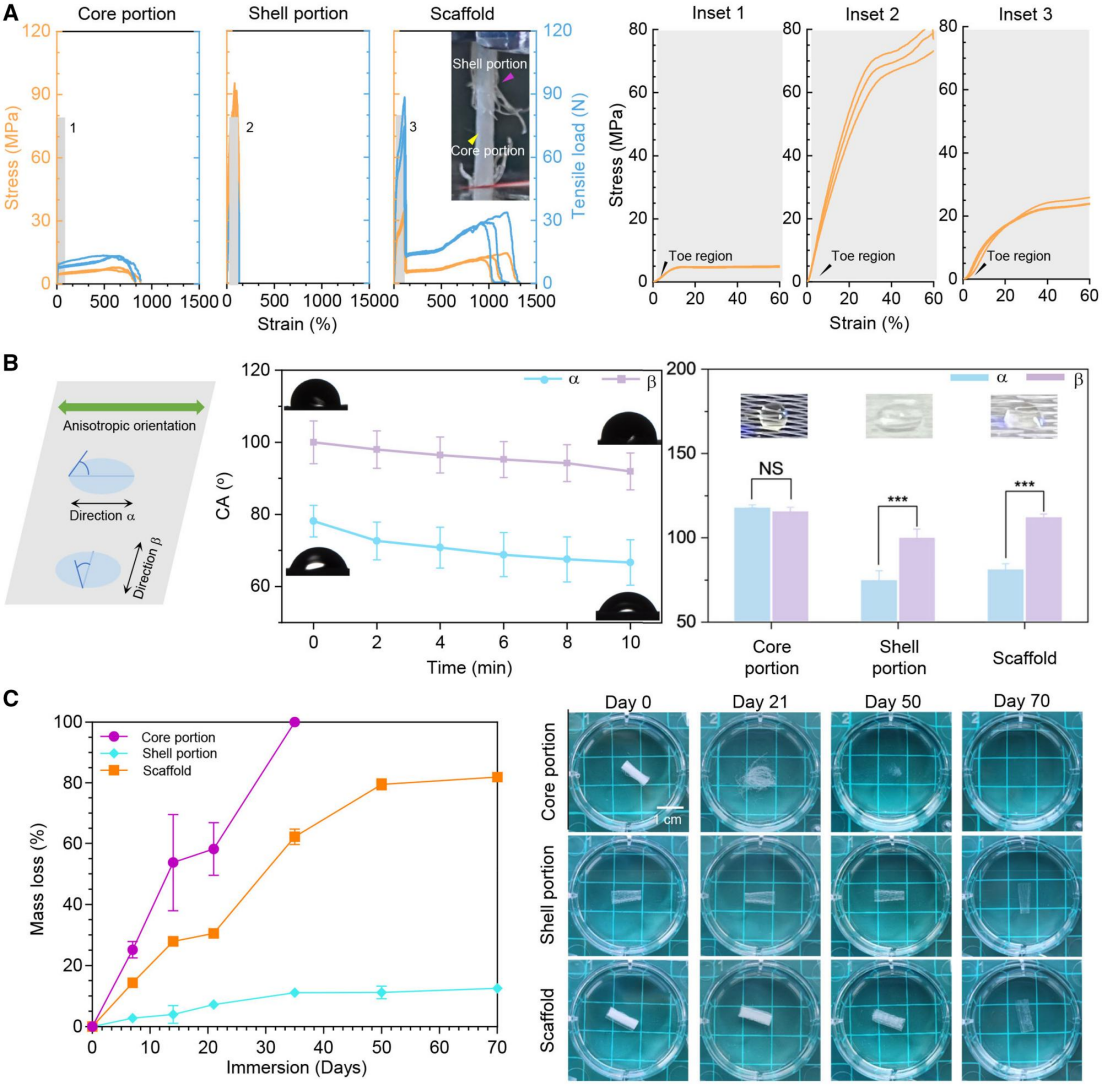
Properties of core-shell tendon scaffold. (**A**) Mechanical properties. Orange colour, stress–strain curves. Blue colour and tensile loading curves. Optical image: scaffold showing a two-stage fracture. Insets 1–3, enlarged elastic regions. (**B**) Surface wettability. Optical images of water droplets. *n* = 3; NS, *P* > 0.05 and ***P < 0.001. (**C**) Mass loss curves and optical images of the scaffold core and shell subjected to *in vitro* accelerated hydrolysis (alkaline solution, 3 mol L^−1^). *n* = 4. Scale bar, 1 cm. CA, contact angle of water.

To assess the compliance of the fabricated core-shell scaffold for tendon applications, Figure 3A presents the tensile stress–strain curves. The scaffold incorporated the characteristics of both the core and shell, resulting in distinct toe and elastic regions, followed by plastic deformation. As the strain increases, the toe region is characterized by a slow, nonlinear rise in stress. This is primarily due to the EHD-printed fibre patterns of the core, where the angled fibres (15°) were able to align gradually, although the shell alone showed a minimal impact on the toe region. The observed toe region, ranging from 1-3% strain, is consistent with that of the natural tendons serving to buffer the abrupt tensile strain applied to the scaffold [13]. In previous studies, curved fibres have been investigated to mimic the mechanical behaviour in the toe region [17]. In comparison, the angled fibres in this study demonstrate a notable resilience, easily returning to their original state after experiencing tensile forces. Owing to their limited loading capability, after alignment, bioinspired fibres are prone to plastic deformation. However, with a reinforced shell, the core fibres can be protected to avoid overloading, thereby significantly enhancing the overall mechanical stability.

In the elastic region, the scaffold demonstrated an intermediate behaviour between that of the core and shell. Due to the reinforcing effect of the shell, the scaffold achieved a yield stress 2.1 times and Young’s modulus 2.4 times greater than those of the core (Table 1). This reinforcing effect is due to strain hardening, which offsets the weakening effect caused by hole enlargement in the shell. Compared to the reported scaffolds featuring core-shell (∼1–8 MPa) [26], braided (∼5–121 MPa) [36] and knitted (∼24–34 MPa) [36] types, the resulting Young’s modulus of the scaffold in this study was superior and approached more that of the human Achilles tendon (217–1200 MPa) [3]. It is also worth noting that the yield stress of the core is ∼5 MPa, above which the EHD-printed fibres alone will be subjected to plastic deformation and lose their elastic properties. In this study, with the core-shell structure, the fabricated scaffold can achieve a higher level of yield stress because of the shielding effect of the shell bearing the most excessive loading and protecting the core from excessive deformation.

**Table 1.** Mechanical properties of the core-shell tendon scaffold (*n* = 3)

Samples	*E* (MPa)	σ (MPa)	ε (%)	Ultimate stress (MPa)	Break strain (%)
Core	54.8 ± 2.8	5.2 ± 1.3	14.7 ± 0.3	7.5 ± 0.6	901.9 ± 50.7
Shell	252.0 ± 15.5	49.1 ± 4.5	19.6 ± 1.1	92.8 ± 2.0	126.1 ± 5.4
Scaffold	130.1 ± 9.8	11.1 ± 1.0	11.3 ± 2.3	32.6 ± 2.2	1230.0 ± 101.2
Human Achilles tendon [3]	∼217–1200	NA	NA	∼21–80	∼7–15%

In contrast to the core, the scaffold with a small strain increase displayed a fast stress increase in plastic deformation (Figure 3A), which is similar to that of the natural tendon [37]. This is attributed to the reinforcing effect of the shell due to its strain-hardening process by uniaxial stretching [17]. Consequently, the scaffold achieved a fracture stress 4.3 times greater than that of the core (Table 1). The ultimate fracture load of the scaffold (1 cm in length and 3 mm in diameter) is 78.5 N, which is larger than the sum of 13.4 N (the core) and 51.1 N (the shell, Supplementary Table S3). This indicated a synergistic reinforcement effect between the core and shell, leading to a scaffold capable of withstanding high tensile loads. Finally, the fabricated scaffold exhibited a two-stage plastic deformation until fracture, corresponding to the preferential deformation in the shell and later deformation in the core.

Figure 3B shows the WCA measured along and across the surface structures of the scaffold. The WCA along the surface geometry was much lower than that across the structures, demonstrating higher surface wettability. This is because of the stretching-induced micro-ridges/grooves. With prolonged contact, the anisotropic wettability was well maintained, indicating improved surface wettability with a decreased WCA. This is probably because the hydroxyl groups in PCL interact with water molecules and facilitate their adsorption on the surface. A comparative study indicated similar surface wettability between the scaffold and shell. In contrast, isotropic surface wettability was observed for the core, and the WCA was much higher than that of the scaffold and shell. As shown in the Figure 3B insets, the water droplet demonstrates an approximately circular shape on the core surface, which may be because it covers multiple fibres and air gaps in all directions. This leads to the isotropic surface wettability observed for the core.

As shown in Figure 3C, the scaffold underwent sequential degradation from the core to the shell. After 21 days of degradation, the core preferentially lost its structural integrity and became entangled fibres. After 50 days of degradation, it completely broke down into debris. In contrast, the shell displayed significantly improved properties against degradation and maintained its structural integrity over the investigated hydrolysis period of 70 days. It is evident that PCL after uniaxial stretching follows surface-controlled degradation, because the improved polymer chain recrystallization and densification make it difficult for erosive liquids to penetrate the polymer surface [38]. In contrast, bulk degradation accounts for the loss of structural integrity, as observed for the spheroid structure in the core. In line with these results, the core exhibited a mass loss rate that was much higher than that of the shell, leading to approximately 100% degradation in the core on day 30 and only approximately 10% degradation in the shell until day 70. The scaffold achieved approximately 60% and 80% mass loss on days 30 and 80, respectively. This is because of the preferential degradation in the core, which provides space for the growth of new tissues. These results demonstrate the prolonged structural support of the scaffold, which compensates for the mechanical weakness of the core and ensures security before new tissue formation of the damaged tendon.

### Cytocompatibility of the tendon scaffold

**Figure 4.**
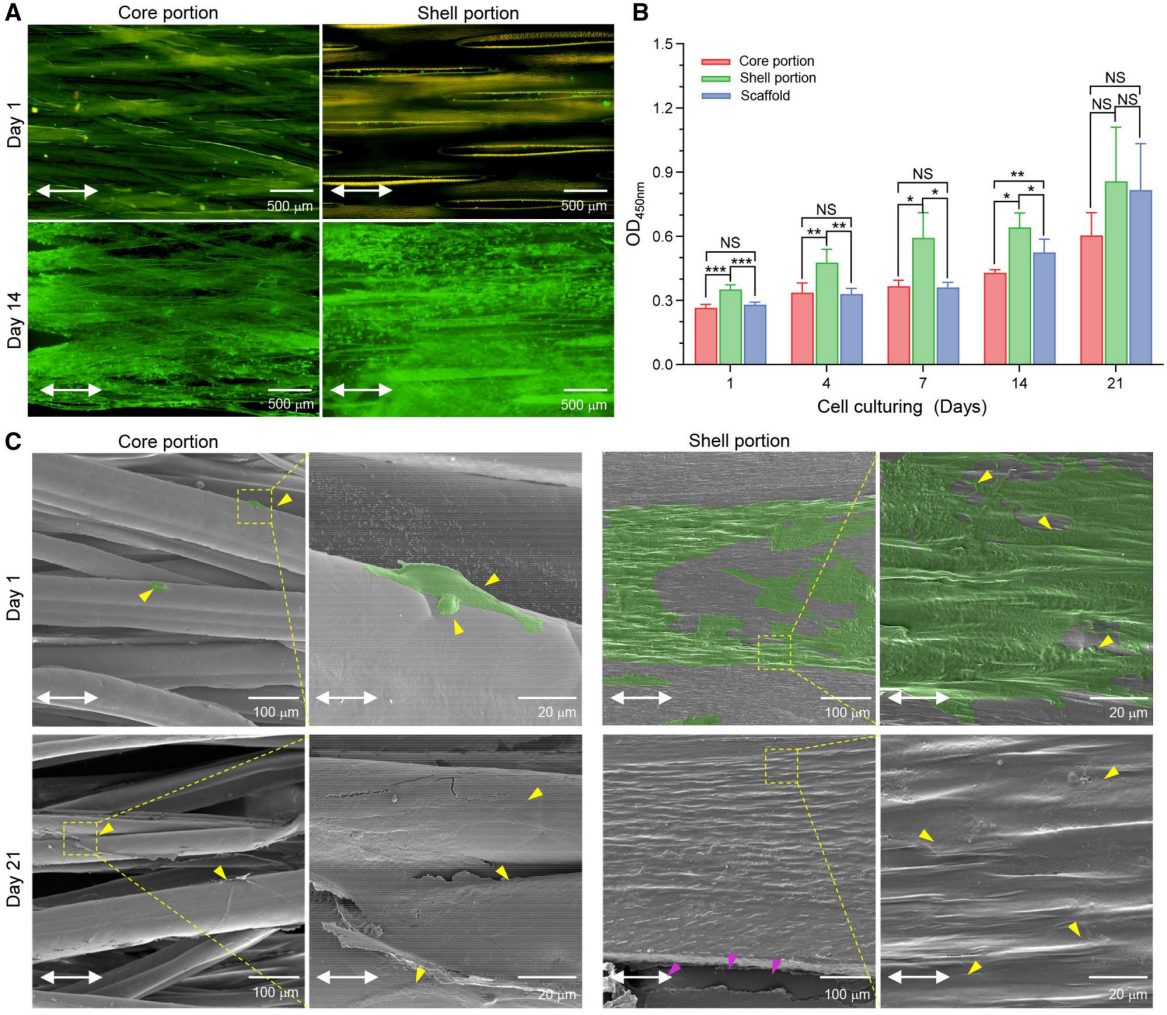
*In vitro* cytocompatibility of the core-shell tendon scaffold. (**A**) CLSM images of live and dead cell staining of MSCs cultured in tendon scaffolds for 1 and 14d. Green: FDA-labelled live cells; red: PI-labelled dead cells. Scale bar, 500 µm. (**B**) Metabolic levels of MSCs cultured in tendon scaffolds (*n* = 5; NS, *P* > 0.05, *P < 0.05, **P < 0.01 and *** *P* < 0.001). (**C**) SEM images of MSCs morphology after culturing the tendon scaffolds for 1 and 21 days. Yellow arrow, cells; green colour, artificially labelled cell shapes; purple arrow, cells in the through-holes; double-headed white arrow, structural direction of the scaffold core and shell. Scale bars, 100 µm (low magnification) and 20 µm (high magnification).

Figure 4A shows fluorescence images of MSCs stained with FDA/PI. The predominance of FDA-labelled live cells was observed on the scaffold’s shell. The porous structure of the shell facilitates cell migration into the core, enabling efficient nutrient exchange and supporting cellular viability. On day 1, some red-labelled dead cells were observed in the scaffold’s core, which may be due to the entrapment of damaged cells during the seeding process. In line with this, after prolonged culture for 14 days, an increased cell population with no dead cells was observed. The details of the cellular proliferation are shown in Figure 4B. Compared to the core, intimal cell growth was enhanced on the shell, which accounted for higher levels of cell proliferation over the investigated period. This is probably because of the high porosity of the core, which causes cell loss during the seeding and subsequent adhesion stages. The scaffold exhibited a trend of cell proliferation similar to that of the core, which was elevated during the later *in vitro* culturing period of day 7–21.

SEM images further indicated that the cells could elongate along the topographical structures of both the core and shell during the very early culture period (day 1, Figure 4C). Pseudopodia were mainly observed at the cell ends. The geometric curvature of individual fibres in the core and ridges in the shell provides contact guidance on each cell, whereas the overall anisotropy of these topographies accounts for the control of the cell alignment in a consistent direction over the scaffold. Prolonged culture results in cell proliferation. In the core, the cells tended to bridge different fibres and penetrate into the space among those fibres, as observed on day 21. On the shell, the cells exhibited nearly 100% coverage of the non-porous surface. In these observations, overall cell alignment was maintained along the surface structures. The cells in the core appeared to have less spreading morphology than those on the shell. Our results demonstrate the proper microenvironment of the tendon scaffold with no obvious cytotoxicity and good cell proliferation.

### Expression of tendon-related genes and proteins in the scaffold

**Figure 5.**
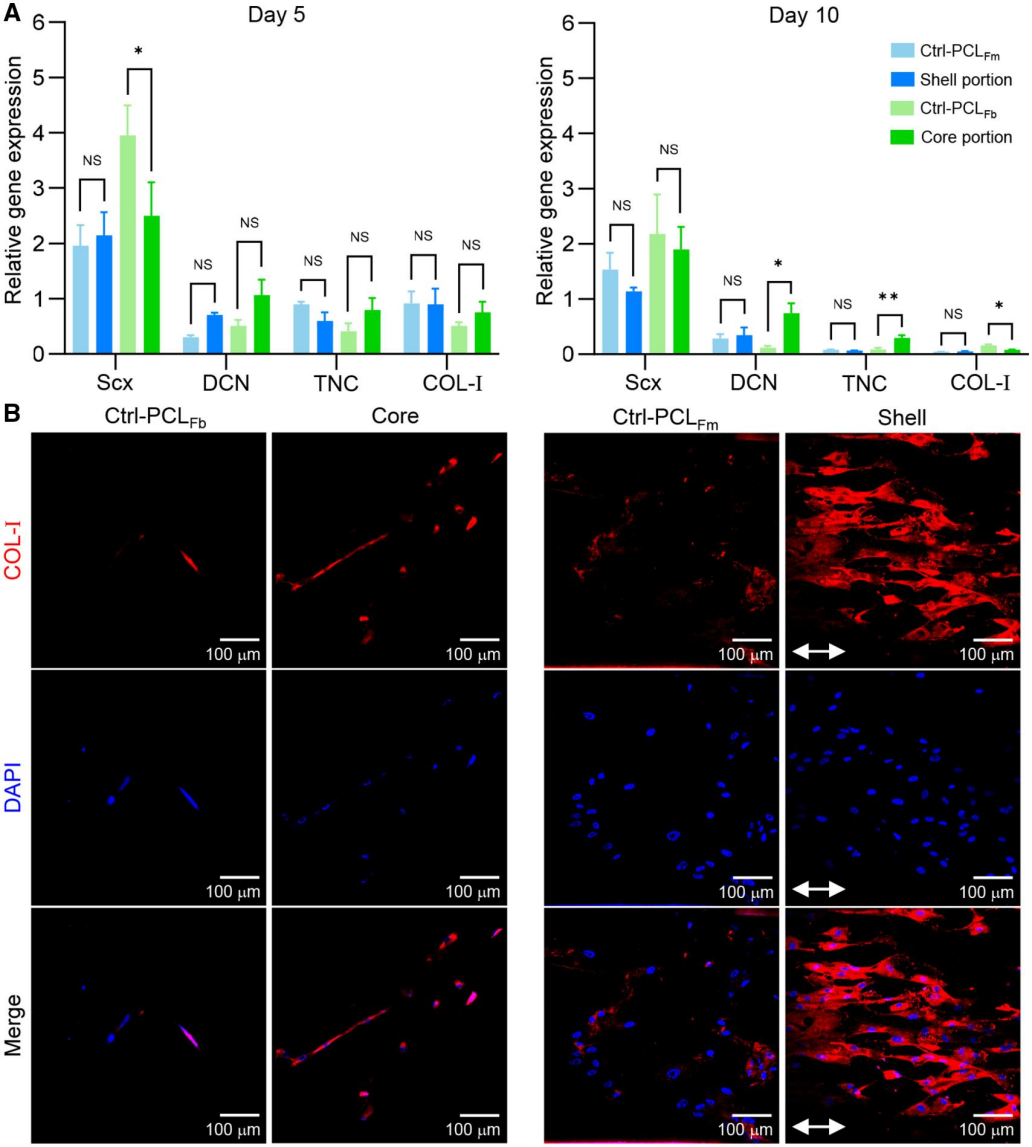
*In vitro* tendon-associated gene and protein expression in human MSCs. (**A**) Genetic expression of Scx, DCN, TNC and COL-I after cell culturing for 5 and 10 days. Ctrl-PCL_Fm_, control group of the scaffold’s shell; and Ctrl-PCL_Fb_, control group of the scaffold’s core (*n* = 3; NS, *P *> 0.05, *P < 0.05 and **P < 0.01). (**B**) CLSM images of the COL-I-labelled cells after 5 days of culturing. Red colour: COL-I and blue colour: DAPI-labelled nucleus DNA. Scale bar, 100 µm.

Figure 5A shows the positive expression of tendon-related genes in human MSCs, including Scx, DCN and TNC corresponding to the early- to middle-term tenogenic markers [13]. Compared to the Ctrl-PCL_Fm_ group, cells cultured for 5 days with the scaffold’s shell showed higher expression levels of Scx and DCN and a lower level of TNC, although significant differences are not observed. In line with the above observations, the scaffold’s core exhibited a lower expression level of Scx in MSCs and higher levels of DCN and TNC compared to those of the Ctrl-PCL_Fb_ group, respectively. Prolonged culturing promoted the gene expression in MSCs, shifting towards the more mature tenogenic markers. The higher expression levels of DCN and TNC were observed on the scaffold’s shell than in its control group. A similar trend was obtained in the scaffold’s core, where the expression levels of DCN and TNC remained higher than those of the control group with statistical differences.

The expression of a major tendon matrix protein COL-I was also investigated, which at the genetic level showed comparable levels in groups of the scaffold’s shell and its control. Differently, the scaffold’s core obtained a higher level of COL-I expression than its control group. With the prolonged culturing of 10 days, the trend on COL-I expression remained unchanged in the scaffold’s shell, while a declined expression level was observed in the scaffold’s core. Figure 5B shows confocal fluorescence images of human MSCs labelled with COL-I and nuclear DNA. It was observed that at the protein level, the cells after 5 days of culturing showed obviously enhanced COL-I expression in both the scaffold’s core and shell compared to their control groups, respectively. Cultured on the scaffold’s components, the cells obtained directional growth toward the geometric anisotropy of the scaffold.

### *In vivo* tendon regeneration via the scaffold

**Figure 6.**
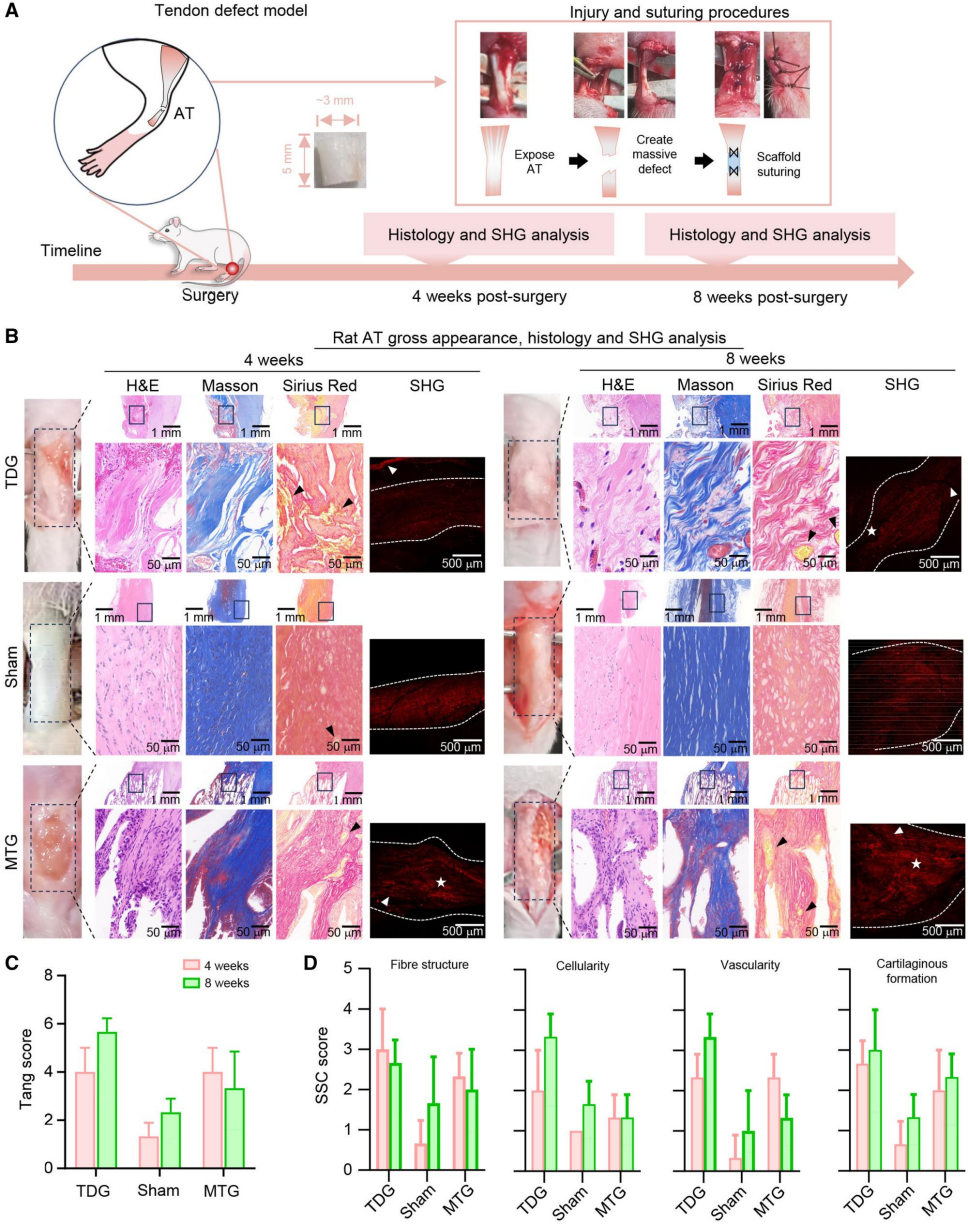
*In vitro* tendon regeneration using a core-shell scaffold. (**A**) Schematic illustrating the rat AT injury model. (**B**) Gross appearance, histological analysis and SHG analysis of Achilles tendon tissue at weeks 4 and 8. Black arrow, adipose tissue; white dotted line, edges of SHG signal distributed region; white arrow in the TDG at week 4, linear structure of abnormally high signal at the tissue interference; white star and arrow in the TDG at week 8, defective tendon regeneration with linear strip in the low-to-non-signal area; white arrow in the MTG at weeks 4 and 8; linear ingrowth of collagen fibre and residual material; and white star in the MTG at weeks 4 and 8, improved signal at the site of suturing. (**C**) Tang score for tendon adhesion (*n* = 3). (**D**) SSC score for tendon regeneration assessment (*n* = 3).

Figure 6A illustrates the animal modelling process. A 5-mm defect was created after exposing the Achilles tendon. In the MTG procedure, the defect was repaired using a scaffold, whereas in the TDG, the defect was repaired via direct suturing. The Sham group underwent no defect creation and served as the positive control group. As shown in Supplementary Figure S3, the scaffold exhibited suitable properties for suturing. Gross observation revealed scar tissue formation at the tendon gapping site in all injury groups, with varying degrees of adhesions, except in the Sham (Figure 6B). Similar tendon adhesion phenomena have been reported previously using ECM scaffolds [39]. However, the extent of scar tissue formation in the MTG was significantly reduced compared to that in the control of TDG, with longitudinal ingrowth of collagen fibres in the scaffold, indicating improved biomechanical properties resembling those of the normal tendon structure.

In terms of peritendinous tissue proliferation, the control group of TDG exhibited more prominent neo-angiogenesis; however, the newly formed vessels intersected with disorganized collagen. In the TDG, fibrosis of the injured tendon site was more pronounced, with the peritendinous tissue appearing rough and resilient, accompanied by significant tendon retraction. This indicates severe adhesion to the surrounding tissue. Peritendinous adhesion in the MTG was significantly reduced, and tendon regeneration was restricted within the implanted scaffold. The Tang score indicates that at week 4, both the TDG and MTG exhibited similar levels of peritendinous adhesion. However, by week 8, tendon adhesion improved in the MTG, whereas tendon adhesion worsened significantly in the TDG (Figure 6C).

Under the microscope, the control group of TDG also exhibited a high degree of disorganized fibre arrangement, with disrupted collagen orientation and larger interfibre gaps (Figure 6B), characteristic of unbenefited tendon remodelling. This was improved with scaffold implantation, where the regenerated collagen fibres were aligned in a pre-designed, linear pattern within the scaffold. Masson’s and Sirius Red staining further confirmed enhanced collagen synthesis, improved fibre arrangement, and increased collagen content within the scaffold. The undegraded material of the scaffold caused porous gaps between the newly generated tendon tissues, suggesting the need for improved degradation capability.

From a cellular perspective, both TDG and MTG showed infiltration of adipose tissue around the newly formed collagen, along with an accumulation of fibroblasts and inflammatory cells. Neo-angiogenesis and cartilaginous formation were most pronounced in the control group and increased as the time post-injury proceeded (Figure 6D). In contrast, the MTG exhibited reduced cellularity along with significantly lower fibrous formation, vascularity and cartilaginous tissue, suggesting that it is a promising approach for Achilles tendon repair. The biomimetic structure of the tendon scaffold supports cellular activities that are essential for tissue regeneration.

Using a two-photon microscope, SHG analysis revealed a regenerated collagen structure at higher resolution and sensitivity (Figure 6B). In the TDG, the newly formed collagen structures appeared thin and disorganized, with several longitudinal defects in the middle section of the tendon at both 4 and 8 weeks. These findings suggest that the tissue has a diminished capacity to withstand mechanical stress. In contrast, the MTG demonstrated distinct longitudinal infiltration of collagen fibres into the tendon defect area within the scaffold at both 4 and 8 weeks. Signal intensity was significantly greater at week 8 compared to both TDG and Sham, suggesting enhanced collagen regeneration. Notably, the signal intensity in the TDG was significantly lower than in the MTG, and the newly formed collagen appeared thicker and more prominent. MGV analysis provided a comprehensive assessment of the collagen content and distribution (Supplementary Figure S4). In the TDG, the MGV was generally lower than in the MTG at both weeks 4 and 8, and became more dispersed by week 8. In contrast, the MTG exhibited lower but more organized MGV in a linear pattern at week 4, which became more pronounced around the scaffold by week 8, suggesting enhanced tendon regeneration and organization facilitated by the scaffold.

One limitation of our present study is the absence of comprehensive biomechanical testing on the *in vivo* scaffold constructs. Tendons are subjected to repetitive stress during movement, making fatigue resistance a critical factor in long-term function and surgical repair success. In addition, suture pull-through resistance is also a critical property of tendon graft, which resists tearing and pulling out of the tissue under tensile forces. Although material characterizations and *in vitro* assessments provided valuable insights of the scaffold’s mechanical properties such as Young’s modulus, elasticity and tensile strength, which are critical for tendon repair applications, future studies are needed to include systematic biomechanical evaluations for more accurately assessing the scaffold's functional performance and its ability to withstand physiological loads *in vivo*. It is also valuable to note that numerous regulations affect the process from DNA transcription to successful deposition of a tendon matrix protein. This results in the varied expression trends of a gene at the transcription and protein levels. Compared to tenogenic growth factors (e.g. bone morphogenetic protein and basic fibroblast growth factor), the remarkable performance of geometric cues as developed in our core-shell scaffold is more likely to induce cell morphogenesis into alignment, thereby resembling the structure and mechanical properties of natural tendon [17]. In our study of tendon regeneration, the tissue adhesion was evaluated by the Tang score. However, adhesions occur when scar tissue forms between a tendon and its adjacent tissues, restricting gliding motion and leading to stiffness, pain, and functional impairment. Therefore, balancing adhesion prevention vs. tendon healing strength remains a clinical challenge.

## Conclusions

This study presents a method for fabricating a core-shell tendon scaffold with a fibrous core that exhibits high porosity and controlled anisotropy, surrounded by a dense, sheet-like shell with micro-ridges and through-hole arrays. These structural features enable sequential core-to-shell degradation, enhanced wettability, and mechanical properties comparable to those of human Achilles tendons. The fabrication process ensures non-cytotoxicity, and *in vivo* studies show that the scaffold promotes tendon regeneration, facilitating matrix fibre alignment and structural organization in a rat Achilles tendon defect model. Our results suggest that this core-shell scaffold method has strong potential for tendon tissue engineering applications.

## Supplementary Material

rbaf088_Supplementary_Data
