# Supplementary material for: Mechanically reinforced core-shell scaffold with integrated structure and function for accelerated tendon repair
Source: Regen Biomater. 2025 Aug 18;12:rbaf088. doi: 10.1093/rb/rbaf088 (PMC12448295; doi:10.1093/rb/rbaf088)
Supplement: rbaf088_Supplementary_Data [file rbaf088_supplementary_data.docx]

Supporting Information

Tables

**Table S1** Primers used for quantitative real-time RT-PCR analysis.

| **Genes** | **5’-3’** | **Primers** |
| --- | --- | --- |
| GAPDH | Forward | AGCCTCAAGATCATCAGCAA |
|  | Reverse | GTCATGAGTCCTGCCACCAT |
| Scx | Forward | CGAGAACACCCAGCCCAAAC |
|  | Reverse | CTCCGAATCGCAGTCTTTCTGTC |
| DCN | Forward | CAGCATTCCTCAAGGTCCT |
|  | Reverse | GAGAGCCATTGTCAACAGCA |
| TNC | Forward | ACTGGCAAGATCACAACAGC |
|  | Reverse | CCCACAATGACTTCCTTGACTG |
| COL-I | Forward | CGAAGACATCCCACCAATACC |
|  | Reverse | GTCACAGATCACGTCATCGC |

**Table S2.** The standards of the Tang Score and the Soslowsky, Svessen and Cook Score (SSC) for evaluating tendon adhesion and regeneration, respectively [1].

| **Tang Score** | **Quantity** | | **Quality** | |
| --- | --- | --- | --- | --- |
| 0 | No adhesion | | No adhesion | |
| 1 | Sparse filaments | | Regular, elongated and thin filaments | |
| 2 | High number of filaments | | Irregular filaments | |
| 3 | Countless filaments | | Dense and non-filamentous adhesions | |
|  | **Degree of adhesion (sum of above)** | | | |
| 0 | No adhesion | | | |
| 2 | Mild adhesion | | | |
| 3,4 | Moderate adhesion | | | |
| 5,6 | Severe adhesion | | | |
| **SSC score** | **Fiber structure** | **Cellularity** | **Vascularity** | **Cartilaginous Formation** |
| 1 | Normal structure with parallel and compact collagen fibers | Tapered and elongated nuclei, not very visible and with little cytoplasm | The few vessels are parallel to the collagen fibers | No cartilaginous formation |
| 2 | Slight changes with separate collagen fibers with increased ripple (<25%) | Increased cell rotundity: nuclei become less oval and rounder with little cytoplasm | Slight increase in the vessels | Isolated hyaline cartilage nodules |
| 3 | Moderate changes with disorganized, separate and disoriented collagen fibers (≥25% and ≤50%) | Increased roundness and cellular size: the nuclei become round with an increase in the cytoplasm | Moderate increase in the vessels | Moderate cartilaginous formations (25–50%) |
| 4 | Marked changes with disorganized and hyalinized collagen fibers (>50%) | The nucleus appears round and wide with abundant cytoplasm and lacuna formation | Marked increase in the vessels | Extended cartilaginous formations (>50%) |

**Table S3**. Mechanical properties of the core-shell tendon scaffold. (n=3)

| **Samples** | **Size** | **Ultimate load (N)** |
| --- | --- | --- |
| Core portion | Length, 1cm; diameter, 3mm | 13.4 ± 0.1 |
| Shell portion | Length, 1cm; diameter, 3mm | 51.1 ± 2.0 |
| Scaffold | Length, 1cm; diameter, 3mm | 78.5 ± 8.6 |

Figures


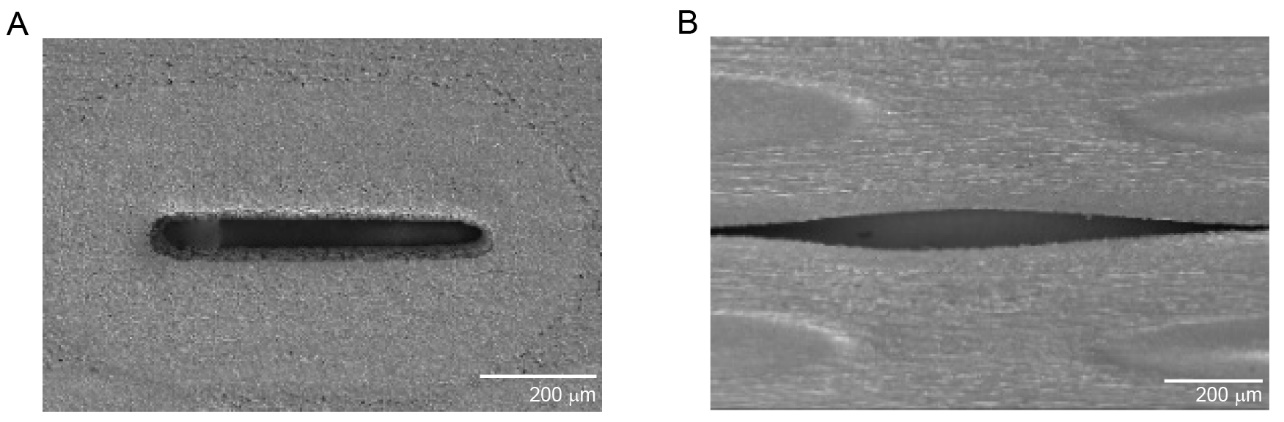


**Figure S1.** Representative SEM images of the through holes. (A) Morphology of the through holes after laser microengineering. (B) Morphology of the through holes after uniaxial stretching.


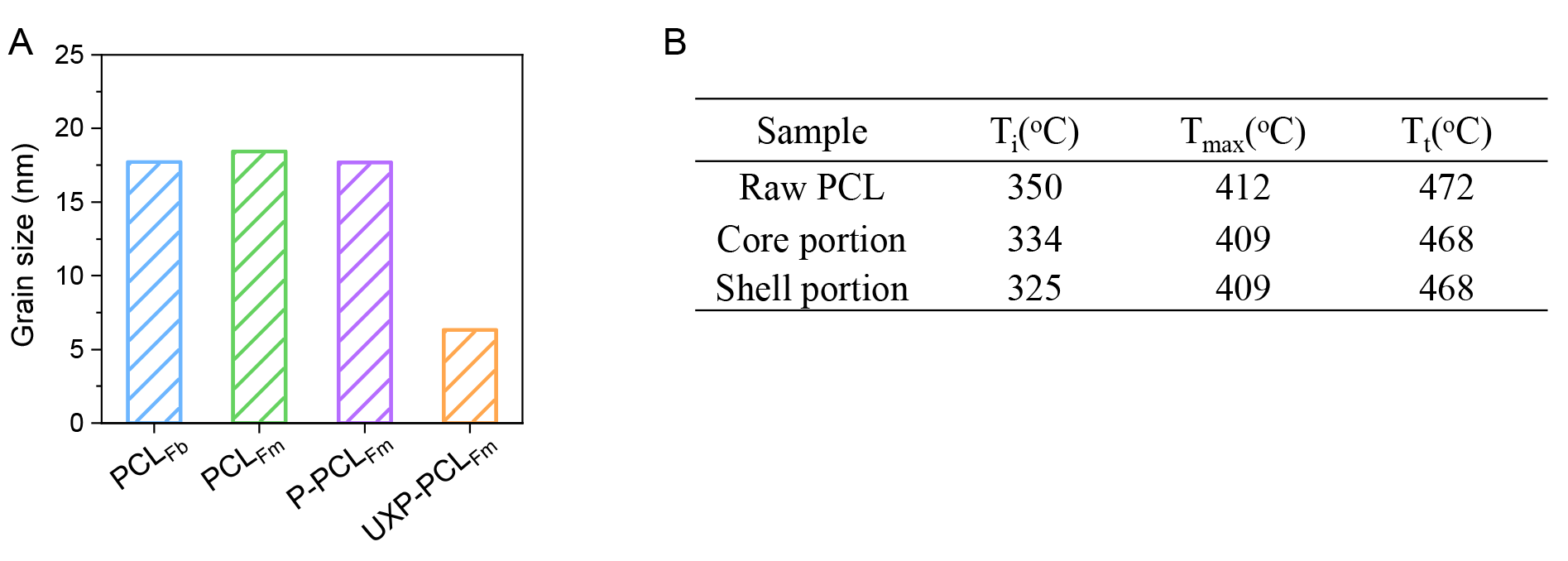


**Figure S2.** (A) Grain size of PCL_Fb_, PCL_Fm_, P-PCL_Fm_ and UXP-PCL_Fm_ calculated from XRD spectra. (B) Parameters related to the thermal decomposition process of TG. T_i_, Initial decomposition temperature; T_max_, maximum decomposition temperature; and T_t_, termination decomposition temperature.


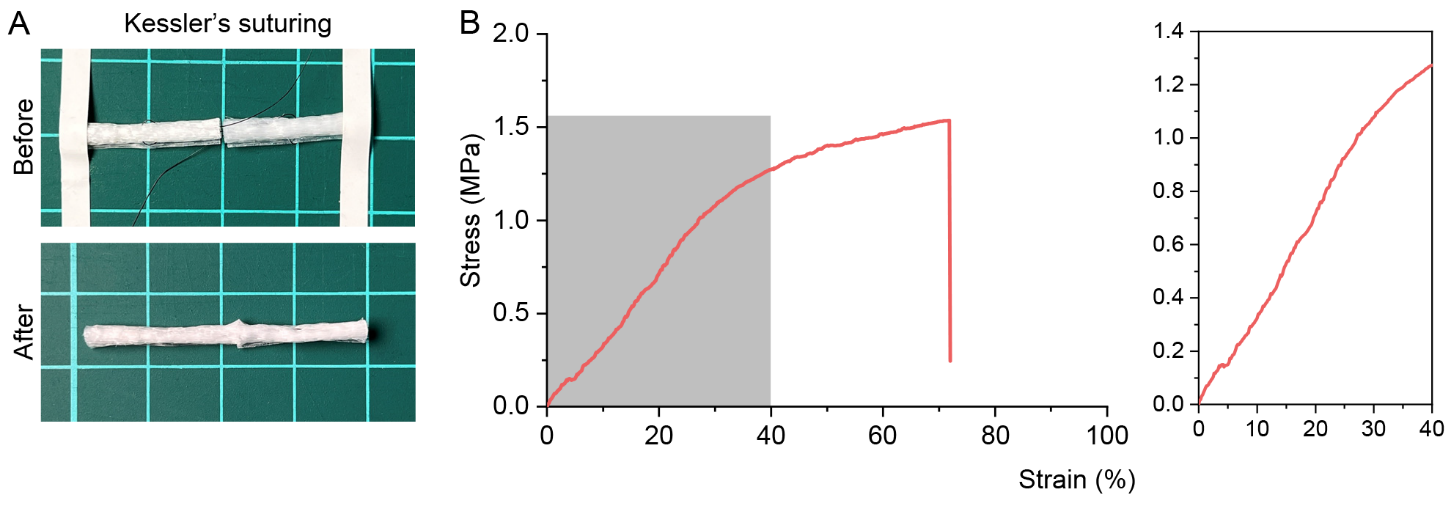


**Figure S3.** Operative capability of the core-shell scaffold. (A) Optical images of the tendon scaffold before (up) and after (down) suturing using the Kessler’s suture method. (B) Representative tensile stress-strain curve of the sutured scaffold.


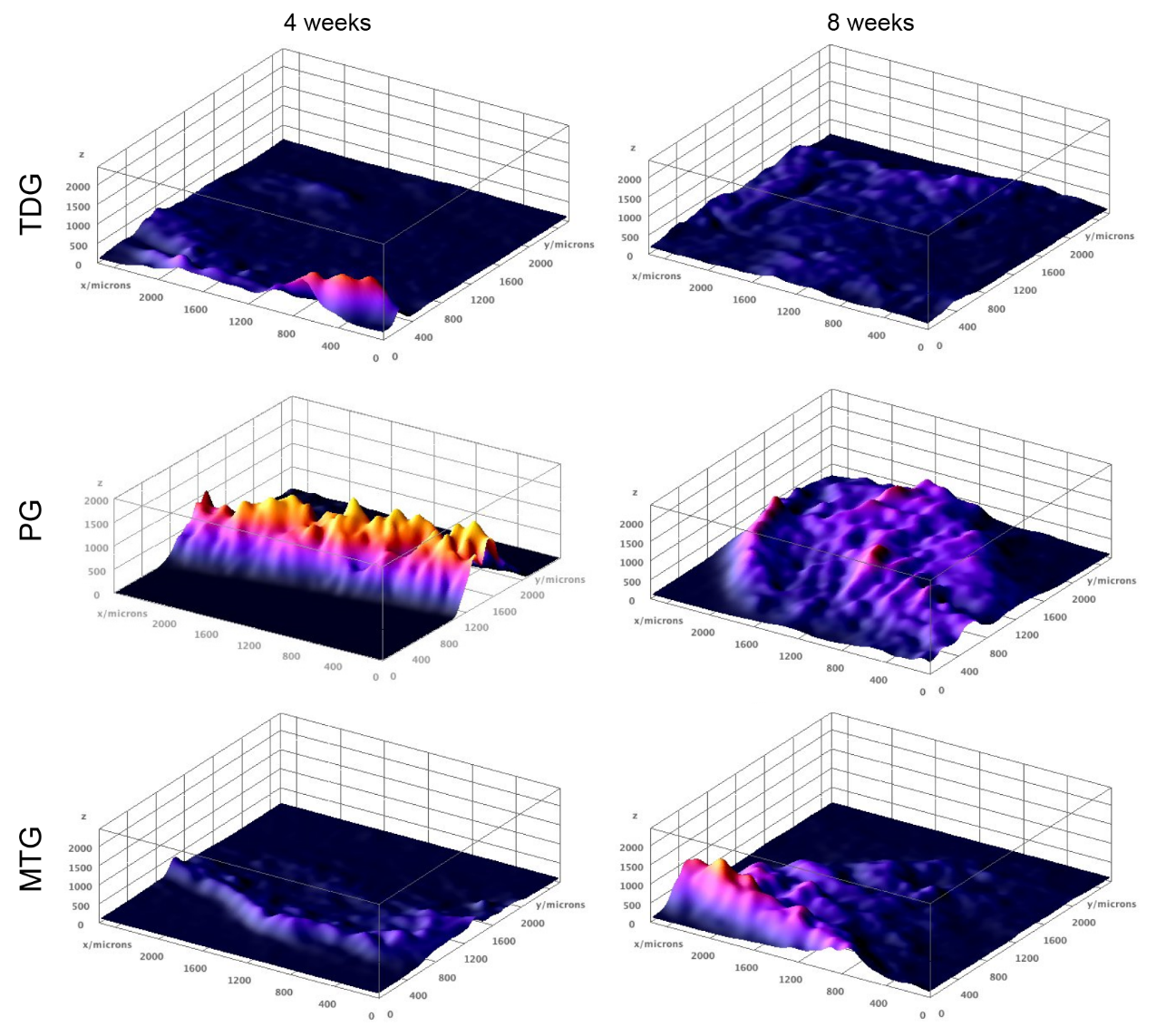


**Figure S4.** The mean gray value of the second-harmonic generation signal. TDG, tendon defect group; PG, pseudosurgery group; MTG, material treatment group.

**Reference**

1. Marchesini A, De Francesco F, Pangrazi PP, Senesi L, Campodonico A, Riccio V, Geuna S, Zavan B, Riccio M. Effectiveness of Hyaluronan Autocross-Linked-Based Gel in the Prevention of Peritendinous Adherence Following Tenolysis. *Applied Sciences* 2021;11:7613.
